# Standardization of Sequencing Coverage Depth in NGS: Recommendation for Detection of Clonal and Subclonal Mutations in Cancer Diagnostics

**DOI:** 10.3389/fonc.2019.00851

**Published:** 2019-09-04

**Authors:** Anna Petrackova, Michal Vasinek, Lenka Sedlarikova, Tereza Dyskova, Petra Schneiderova, Tomas Novosad, Tomas Papajik, Eva Kriegova

**Affiliations:** ^1^Department of Immunology, Faculty of Medicine and Dentistry, Palacky University and University Hospital, Olomouc, Czechia; ^2^Department of Computer Science, Faculty of Electrical Engineering and Computer Science, Technical University of Ostrava, Ostrava, Czechia; ^3^Department of Hemato-Oncology, Faculty of Medicine and Dentistry, Palacky University and University Hospital, Olomouc, Czechia

**Keywords:** next-generation sequencing, variant allele frequency (VAF), coverage depth calculator, sequencing error, small subclones, *TP53* gene

## Abstract

The insufficient standardization of diagnostic next-generation sequencing (NGS) still limits its implementation in clinical practice, with the correct detection of mutations at low variant allele frequencies (VAF) facing particular challenges. We address here the standardization of sequencing coverage depth in order to minimize the probability of false positive and false negative results, the latter being underestimated in clinical NGS. There is currently no consensus on the minimum coverage depth, and so each laboratory has to set its own parameters. To assist laboratories with the determination of the minimum coverage parameters, we provide here a user-friendly coverage calculator. Using the sequencing error only, we recommend a minimum depth of coverage of 1,650 together with a threshold of at least 30 mutated reads for a targeted NGS mutation analysis of ≥3% VAF, based on the binomial probability distribution. Moreover, our calculator also allows adding assay-specific errors occurring during DNA processing and library preparation, thus calculating with an overall error of a specific NGS assay. The estimation of correct coverage depth is recommended as a starting point when assessing thresholds of NGS assay. Our study also points to the need for guidance regarding the minimum technical requirements, which based on our experience should include the limit of detection (LOD), overall NGS assay error, input, source and quality of DNA, coverage depth, number of variant supporting reads, and total number of target reads covering variant region. Further studies are needed to define the minimum technical requirements and its reporting in diagnostic NGS.

## Introduction

Next-generation sequencing (NGS) has rapidly expanded into the clinical setting in haemato-oncology and oncology, as it may bring great benefits for diagnosis, selection of treatment, and/or prognostication for many patients (1). Recently, several articles about the validation of deep targeted NGS in clinical oncology were published (2, 3), including a comprehensive recommendation by the Association for Molecular Pathology and the College of American Pathologists (1). However, the lack of standardization of targeted NGS methods still limits their implementation in clinical practice (4).

One challenge in particular is the correct detection of mutations present at low variant allele frequencies (VAF) and standardization of sequencing coverage depth (1, 5, 6). This is especially important for mutations that have clinical impacts at subclonal frequencies (1) such as the case of *TP53* gene mutations (*TP53*mut) in chronic lymphocytic leukemia (CLL) (7, 8). *TP53* aberrations (*TP53*mut and/or chromosome 17p deletion) are among the strongest prognostic and predictive markers guiding treatment decisions in CLL (9). Nowadays, the European Research Initiative on Chronic Lymphocytic Leukemia (ERIC) recommends detecting *TP53*mut with a limit of detection (LOD) of at least 10% VAF (10), and a growing body of evidence exists dedicated to the clinical impact of small *TP53* mutated subclones in CLL (7, 8).

Sanger sequencing and deep targeted NGS are currently the techniques most used for *TP53*mut analysis (10) as well as for analysis of other genes with clinical impacts at low allele frequencies. Although Sanger sequencing provides a relatively accessible sequencing approach, it lacks the sensitivity needed to detect subclones due to its detection limit of 10–20% of mutated alleles (10). NGS-based analysis has thus gained prominence in diagnostic laboratories for the detection of somatic variants and various technical developments of error correction strategies, both computational and experimental, are being developed for the accurate identification of low-level genetic variations (11). We therefore address the importance of the correct determination of sequencing depth in diagnostic NGS in order to obtain a confident and reproducible detection, not only of low VAF variants. Finally, we performed a dilution experiment to confirm our theoretical calculations, and we close by discussing our experience with diagnostic detection of *TP53*mut in CLL patients and further perspectives about NGS standardization in cancer diagnostics.

## NGS Sequencing Depth and Error Rate

NGS sequencing depth directly affects the reproducibility of variant detection: the higher the number of aligned sequence reads, the higher the confidence to the base call at a particular position, regardless of whether the base call is the same as the reference base or is mutated (1). In other words, individual sequencing error reads are statistically irrelevant when they are outnumbered by correct reads. Thus, the desired coverage depth should be determined based on the intended LOD, the tolerance for false positive or false negative results, and the error rate of sequencing (1, 11).

Using a binomial distribution, the probability of false positive and false negative results for a given error rate as well as the intended LOD can be calculated, and the threshold for a variant calling for a given depth can be estimated (1). For example, given a sequencing error rate of 1%, a mutant allele burden of 10%, and a depth of coverage 250 reads, the probability of detecting 9 or fewer mutated reads is, according to the binomial distribution, 0.01%. Hence, the probability of detecting 10 or more mutated reads is 99.99% (100–0.01%), and the threshold for a variant calling can be defined. In other words, a coverage depth of 250 with a threshold of at least 10 mutated reads will have a 99.99% probability that 10% of the mutant allele load will not be missed by the variant calling (although it can be detected in a different proportion). In this way, the risk of a false negative result is greatly minimized. On the other hand, the probability of false positives heavily depends on the sequencing error rate (as the accuracy of all analytical measurements depends on the signal-to-noise ratio) (1, 11). In our example, the probability of a false positive result is 0.025%; however, the rate of false positives is not negligible when decreasing the LOD to the value close to the error rate. Conventional intrinsic NGS error rates range between 0.1 and 1% (Phred quality score of 20–30) (1, 11) depending on the sequencing platform, the GC content of the target regions (12), and the fragment length, as shown in Illumina paired-end sequencing (13). Therefore, the detection of variants at VAFs <2% is affected by a high risk of a false positive result, regardless of the coverage depth. It is also important to mention that the sequencing error rate applies only for errors produced by sequencing itself and does not include other errors introduced during DNA processing and library preparation, particularly during amplification steps, which further increase error rates (1, 11).

## Minimum Sequencing Coverage in Clinical Settings

**Figure 1:**
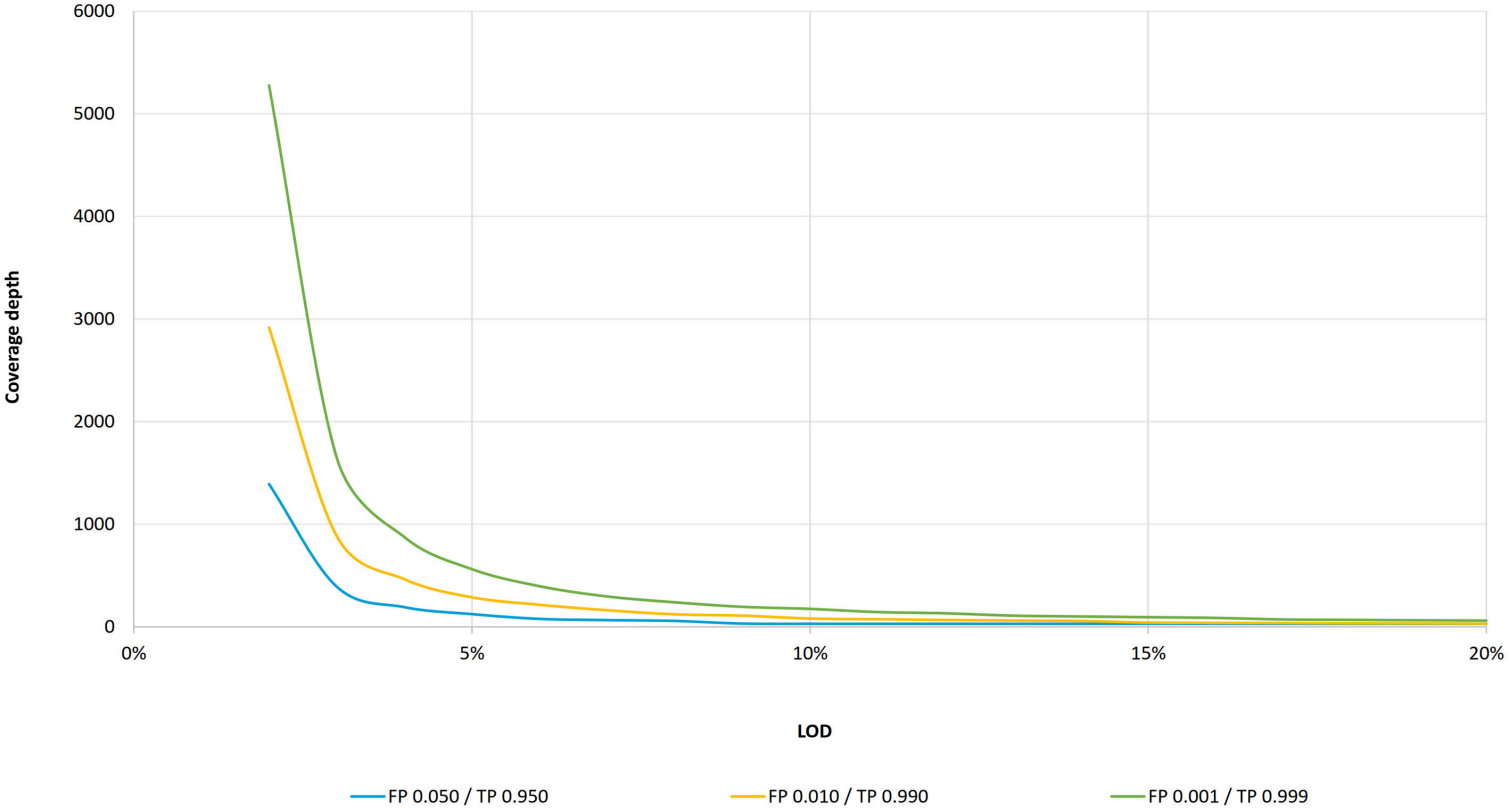
LOD as a function of coverage depth according to the binomial distribution. Coverage depth needed to maintain an intended LOD (within 3–20% VAF range) for three cumulative probability settings: for false positive probability of 0.001 and true positive of 0.999, a LOD of 20% is achieved at 61 coverage depth, a LOD of 10% at 175, a LOD of 5% at 562, and a LOD of 3% at 1,650. For the false positive probability of 0.010 and true positive of 0.990, a LOD of 20% is achieved at 31, a LOD of 10% at 81, a LOD of 5% at 288, and a LOD of 3% at 886 coverage depth, respectively. For the false positive probability of 0.050 and true positive of 0.950, a LOD of 20% is achieved at 30, a LOD of 10% at 30, a LOD of 5% at 124, and a LOD of 3% at 392 coverage depth, respectively.

There is currently no consensus on the minimum required coverage in a clinical setting using deep targeted resequencing by NGS, and so each laboratory has to set its own parameters in order to meet sufficient quality (1, 5). To date, only a few studies have recommended the minimum coverage criteria for deep targeted NGS in clinical oncology: 500 depth of coverage and a LOD of 5% (2), 300–500 depth of coverage without defying the LOD (3), 250 depth and a LOD of 5% with threshold adjustment to 1,000 depth of coverage is required in cases of heterogeneous variants in low tumor cellularity samples (1), and 100 depth with at least 10 variant reads and a LOD of 10% (10). According to the binominal data distribution, a coverage depth of 250 should indeed be sufficient to detect 5% VAF with a threshold of variant supporting reads ≥5 (Figure 1). On the other hand, NGS analysis with a coverage depth of 100 along with a requirement of at least 10 variant supporting reads as recommended by the ERIC consortium (10) would result in a false negative of 45% for samples with a LOD of 10%. To confirm these theoretic calculations, we performed two independent dilution experiments to estimate the performance of *TP53* NGS analysis to detect 10% VAF at a depth of coverage of 100 reads. Indeed, we detected 30% of false negatives (5 positive samples of 7 true-positive samples and 9 positive samples of 13 true-positive samples) in two independent sequencing runs. Unfortunately, the false negative rate is often underestimated in targeted resequencing. Also, a recent study investigating inter-laboratory results of somatic variant detection with VAFs between 15 and 50% in 111 laboratories with reported LODs of 5–15% (6) shows that major errors in diagnostic NGS may arise from false negative results, even in samples with high mutation loads (6). Of three concurrent false positive results, all variants were correctly detected but mischaracterised (6). Since laboratories have not been asked to report coverage depth for other regions than the identified variants (6), we may only assume that low coverage or high variant calling thresholds contributed to the false negative results. These results further highlight the need for standardized coverage depth parameters in diagnostic NGS, taking into account sequencing errors as well as assay-specific errors.

## Frequency of *TP53* Subclonal Mutations in CLL Detected Through Diagnostic NGS

In order to evaluate the occurrence of low VAF in real-world settings, we reviewed our cohort of CLL patients examined for *TP53*mut in our diagnostic laboratory. The *TP53*mut were assessed as reported previously (14, 15). Briefly, *TP53* (exons 2–10 including 2 bp intronic overlap, 5′ and 3′UTR) was analyzed using 100 ng gDNA per reaction. Amplicon-based libraries were sequenced as paired-end on MiSeq (2x151, Illumina) with minimum target read depths of 5,000x. The LOD of *TP53*mut was set up to 1%, and the variants in the range 1–3% were confirmed by replication. Written informed consent was obtained from all the patients who were enrolled in accordance with the Helsinki Declaration, and the study was approved by the local ethical committee.

Of the diagnostic cohort of 859 CLL patients (April 2016–April 2019), 25% (215/859) were positive for *TP53*mut, and of those, 52.6% (113/215) carried variants with VAF at 10% or lower. In line with our observations, a recent study (8) reported the presence of 63 and 84% low burden (Sanger negative) *TP53*muts in CLL patients at the time of diagnosis and at the time of treatment, respectively, and confirmed the negative impact on the overall survival of *TP53*muts above 1% VAF at the time of treatment (8).

## Calculator for Diagnostic NGS Settings for Detection of Subclonal Mutations

**Figure 2:**
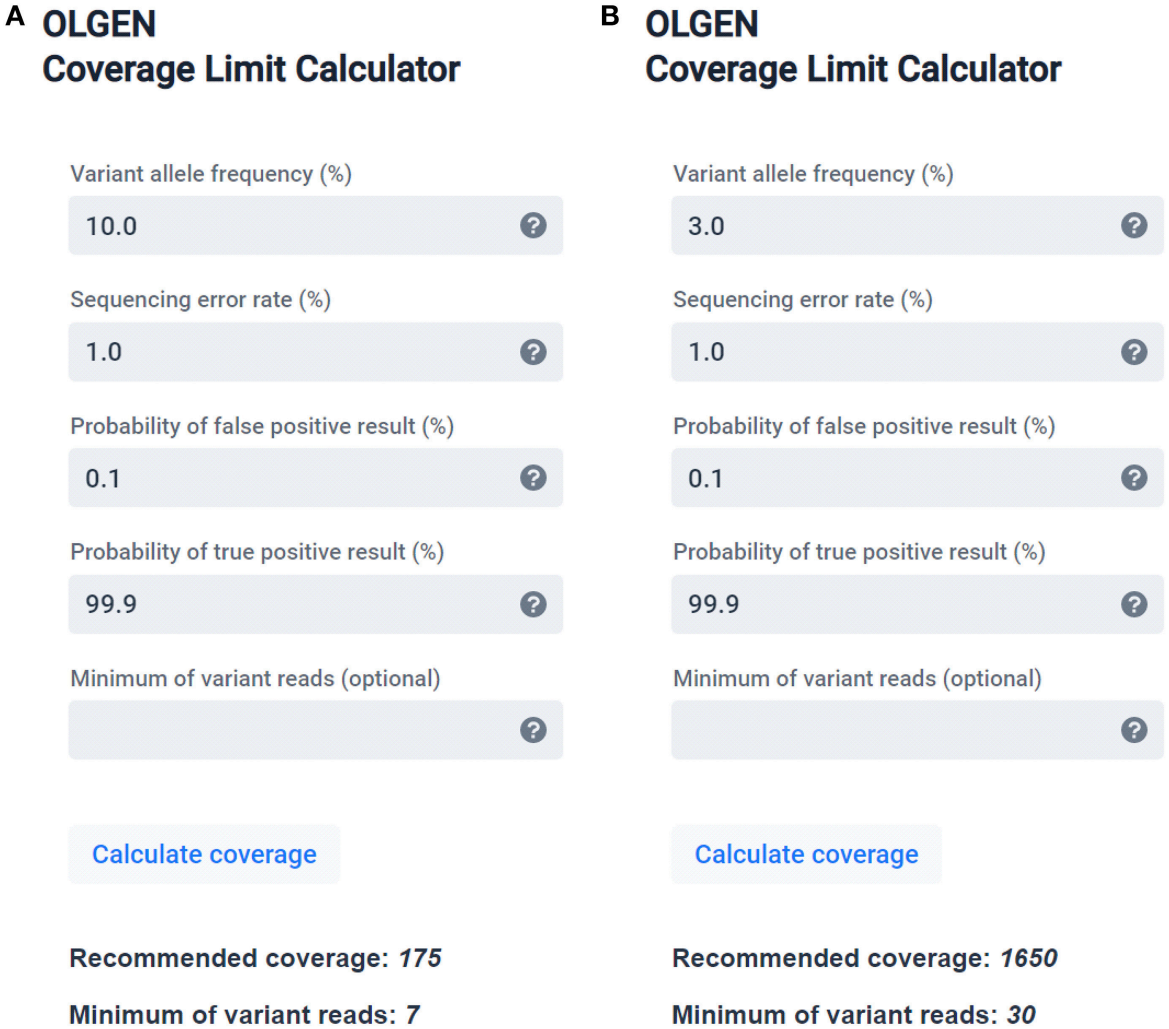
OLGEN Coverage Limit calculator—a simple theoretical calculator suitable for determining the correct sequencing depth and corresponding minimum number of variant reads according to the binomial distribution for a given sequencing error rate and intended LOD recommended for diagnostic NGS. Examples of calculated sequencing depths and the corresponding minimum number of variant reads recommended for variants with **(A)** 10% VAF and 99.9% probability of detection and **(B)** 3% VAF and 99.9% probability of detection.

To assist laboratories with the determination of the minimum proper coverage parameters, we are providing a simple, user-friendly theoretical calculator (software) based on the binomial distribution (Figure 2), described in the Supplementary File. A web (or desktop) application and stand-alone source codes in R are accessible on Github: https://github.com/mvasinek/olgen-coverage-limit. Using this calculator, the correct parameters of sequencing depth and the corresponding minimum number of variant reads for a given sequencing error rate and intended LOD can easily be determined. Moreover, users can also take into account other errors by simply adding assay-specific errors to the sequencing error rate and using this overall error as an input to the calculator. For example, in our case of *TP53* mutational analysis we calculated with the overall error of ~1.16%, thus we set up our minimum coverage depth requirements to 2,000 with threshold of minimum 40 reads for 3% VAF.

## Discussion

Although diagnostic NGS has gained prominence in clinical settings for the assessment of somatic mutations in cancer, insufficient standardization of sequencing parameters still limits its implementation in clinical practice (1), mainly for variants present at low allele frequencies (4). We, therefore, addressed the technical question of correctly determining the sequencing depth in diagnostic NGS in order to obtain confident and reproducible detections of low VAF variants. In particular, we performed theoretical calculations to determine the optimum depth of coverage for the desired probability of detection of variants at low allele frequencies, taking into account the sequencing error rate. Moreover, we confirmed these theoretical calculations by conducting dilution experiments. Based on these observations, we recommend a depth of coverage of 1,650 or higher (together with the respective threshold of at least 30 mutated reads) to call ≥3% variants to achieve a 99.9% probability of variant detection, using the conventional NGS sequencing error only. Variants in the 1–3% VAF range can only be called if the obtained sequence data is of high quality (average Q30 > 90%) and/or when the variants are confirmed by replication or the orthogonal method (1, 11, 16). We are also providing a simple, user-friendly theoretical calculator (software) to assist laboratories with resolving the correct sequencing depth and the corresponding minimum number of variant reads while taking into account the sequencing error rate. Our simple calculator may help to minimize the false positive and false negative results in diagnostic NGS.

Nevertheless, correct sequencing depth is also influenced by assay-specific factors (1). Errors can occur at many stages during DNA processing and library preparation. The most common are amplification errors introduced during NGS library preparation (1, 12, 17). Other common sources of errors have to do with library complexity (the number of independent DNA molecules analyzed), DNA quality, and target region complexity etc. All potential assay-specific errors should be addressed through test design, method validation, and quality control.

Currently, emerging error correction strategies, both computational and experimental, are being developed in order to mitigate the high error rates in diagnostic NGS (11). So far, among the most promising error correction methods are UMI (Unique Molecular Identifiers), which correct for PCR errors (18), and signal-to-noise correction approaches (11). These advances attempt to reduce the LOD, thereby increasing sequencing accuracy needed for future opportunities in NGS diagnosis.

In order to improve the standardization in diagnostic NGS, the estimation of correct coverage depth is a recommended starting point when assessing thresholds surrounding a particular NGS assay. Nevertheless, there is still lack of published guidance regarding the minimum technical requirements and its reporting in NGS, particularly important in detection of clonal and subclonal mutations in cancer diagnostics. This is mainly due to the broad range of library preparation approaches, and numerous variables playing a role in each specific NGS assay, that are difficult to standardize, together with inter-laboratory variability. Therefore, the definition of minimum technical requirements and its reporting in NGS is highly desirable. Based on our experience in diagnostic NGS in haemato-oncology, we suggest to report at least following technical parameters: LOD, overall error of NGS assay (or at least sequencing error rate), the amount of DNA input, source, and quality of DNA, minimum coverage depth and the percentage of targeted bases sequenced at this minimum depth, total number of target reads covering variant region and number of reads supporting the variant. Special emphasis should be given to NGS standardization of the formalin-fixed paraffin-embedded (FFPE) samples (19, 20).

Taken together, our study highlights the importance of correct sequencing depth and the minimum number of reads required for reliable and reproducible detection of variants with low VAF in diagnostic NGS. The calculation of correct sequencing depth for a given error rate using our user-friendly theoretical calculator (software) may help to minimize the false positive and false negative results in diagnostic NGS, in situations related to subclonal mutations among others. The rigorous testing and standardized minimum requirements for diagnostic NGS is particularly desirable to ensure correct results in clinical settings.

## Data Availability

The datasets generated for this study are available on reasonable request to the corresponding author.

## Author Contributions

AP and EK designed the study, interpreted the results, and wrote the manuscript. AP, LS, TD, and PS performed NGS analysis. TP collected the patient samples and clinical data. MV performed bioinformatics analysis and wrote the calculator code. TN prepared web application. All authors read and approved the final version of manuscript.

### Conflict of Interest Statement

The authors declare that the research was conducted in the absence of any commercial or financial relationships that could be construed as a potential conflict of interest.
