# Supplementary material for: Standardization of Sequencing Coverage Depth in NGS: Recommendation for Detection of Clonal and Subclonal Mutations in Cancer Diagnostics
Source: Front Oncol. 2019 Sep 4;9:851. doi: 10.3389/fonc.2019.00851 (PMC6738196; doi:10.3389/fonc.2019.00851)
Supplement: Supplementary file 1 [file Data_Sheet_1.pdf]

SUPPLEMENTARY FILE

Standardization of Sequencing Coverage Depth in NGS: Recommendation for Detection of Clonal and Subclonal Mutations in Cancer Diagnostics

Petrackova Anna, Vasinek Michal, Sedlarikova Lenka, Dyskova Tereza, Schneiderova Petra, Novosad Tomas, Papajik Tomas, Kriegova Eva

Introduction

The OLGEN Coverage Limit software provides an easy way how to estimate the minimal depth of sequencing coverage for given variant allele frequency (v_f), sequencing error (p_e) and limits for cumulative probability of S_E or more errors (p_{fp}) resp. $\hat{v_f}$ or more reads supporting variant allele (p_{tp}) (Figure S1, Table S1).

The computational model assumes that errors are accumulated in one variant allele only, either yielding a detection of false positive variant allele or suppressing detection of variant allele (false negative).

To estimate the minimal required depth of coverage, the software searches for a minimal value in which the cumulative distribution function (cdf) of binomial distribution meets limiting conditions.

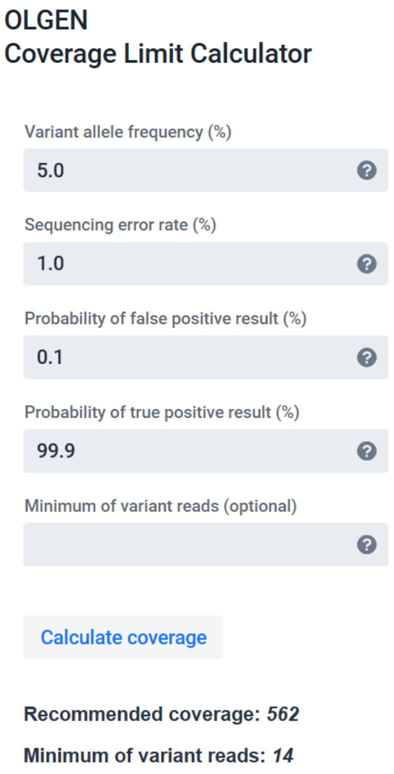

Figure S1: Screen from OLGEN Coverage Limit online application - representative example.

Field	Description/Hint
Variant allele frequency (%)	Intended limit of detection of the NGS assay. Expressed as a percentage of sequence reads carrying a mutant allele.
Sequencing error rate (%)	Intrinsic NGS error rate (usually 0.1-1.0% = phred quality score of 20-30). Must not exceed variant allele frequency. Considering also the assay-specific error, use the sum of assay-specific error and sequence error as input.
Probability of false positive result (%)	Given the sequencing coverage depth, this parameter determines the largest number of false variant reads within a limit given by cumulative probability of a false positive result based on the sequencing error rate.
Probability of true positive result (%)	Given the sequencing coverage depth, this parameter determines the smallest number of true variant reads within a limit given by cumulative probability of a true positive result based on the intended variant allele frequency.
Minimum variant reads (optional)	Minimum number of sequencing reads supporting variant.

Table S1: Description of OLGEN Coverage Limit parameters.

Statistical Model of Minimal Sequencing Coverage

We assume that function $binom(N, p, k)$ returns probability of obtaining exactly k positive instances from N trials with probability of success p. Function $cdfbinom(N, p, k)$ returns cumulative probability of obtaining up to k positive instances from N trials with probability of success p.

Sequencing Error

We assume the sequencing error p_e is distributed binomially. Thus, a probability density function can be plotted for given sequencing error p_e and the number of reads N. An example of probability density function for $p_e = 0.01$ and $N = 200$ is demonstrated in Figure S2. In this example, sequencing errors $SE = 2$ are obtained with the highest probability. This corresponds to the expected value $E[SE] = p_e N = 2$.

Next, we search for the number of sequencing errors SE so that the probability obtained for at most SE errors is at least p_{fp}, i.e. $p(X \leq SE) \geq p_{fp}$. An example of this case is shown in Figure S3 for $p_e = 0.01$, $N = 200$ and $p_{fp} = 0.01$. In this case, the limiting number of sequencing errors is $SE = 6$ reads.

Variant Allele Reads

We assume the variant allele is distributed binomially. If the true frequency of variant allele is v_f and the measured frequency is $\hat{v_f}$ then the probability that we observe $\hat{V_f}$ variant allele reads is given by $p(X = \hat{V_f}) = binom(N, v_f, \hat{V_f})$. Here we used capital letters to distinguish absolute number of variant allele reads from the relative number of variant allele reads. The cumulative probability is $p(X \geq \hat{V_f}) = 1 - cdfbinom(N, v_f, \hat{V_f} - 1)$.

In Figure S4 there are three distributions for three different variant allele frequencies visualized. Vertical lines denote the limit where the cumulative probability $P(X \geq \hat{V_f}) \geq 0.99$.

Taken together, there are three conditions that must apply simultaneously in order to calculate minimal coverage and a minimal number of variant reads to ensure required probability of variant detection:

- $\hat{V_f} > S_E$
- $p(X > E) = 1 - cdfbinom(N, v_f, E) \leq p_{fp}$
- $p(X \geq \hat{V_f}) = cdfbinom(N, v_f, \hat{V_f}) \geq p_{tp}$

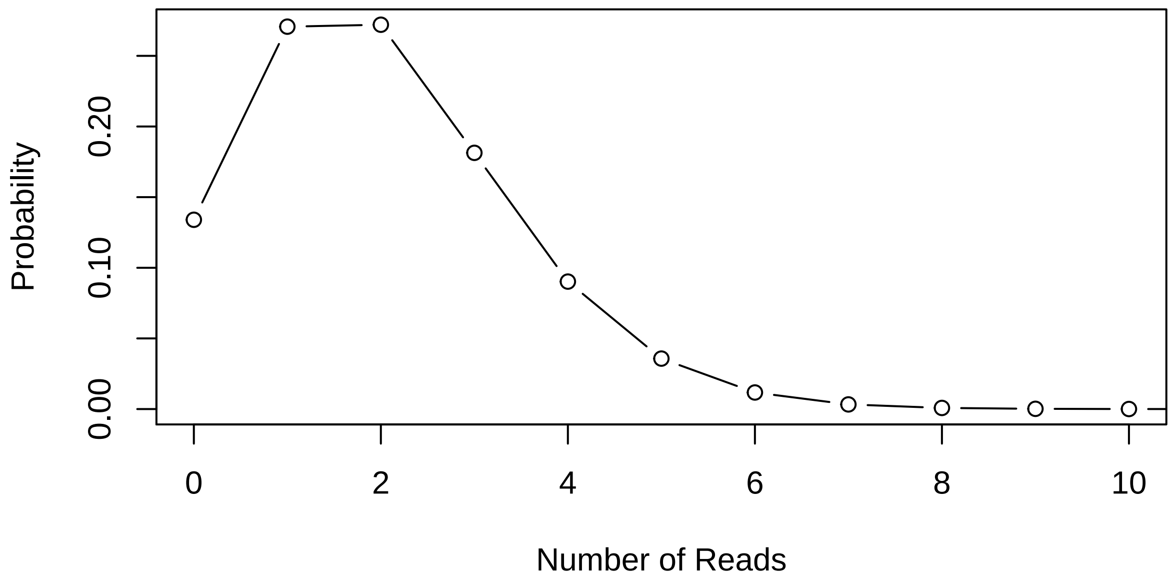

Figure S2: Distribution of sequencing errors for $p_e = 0.01$ and number of reads $N = 200$.

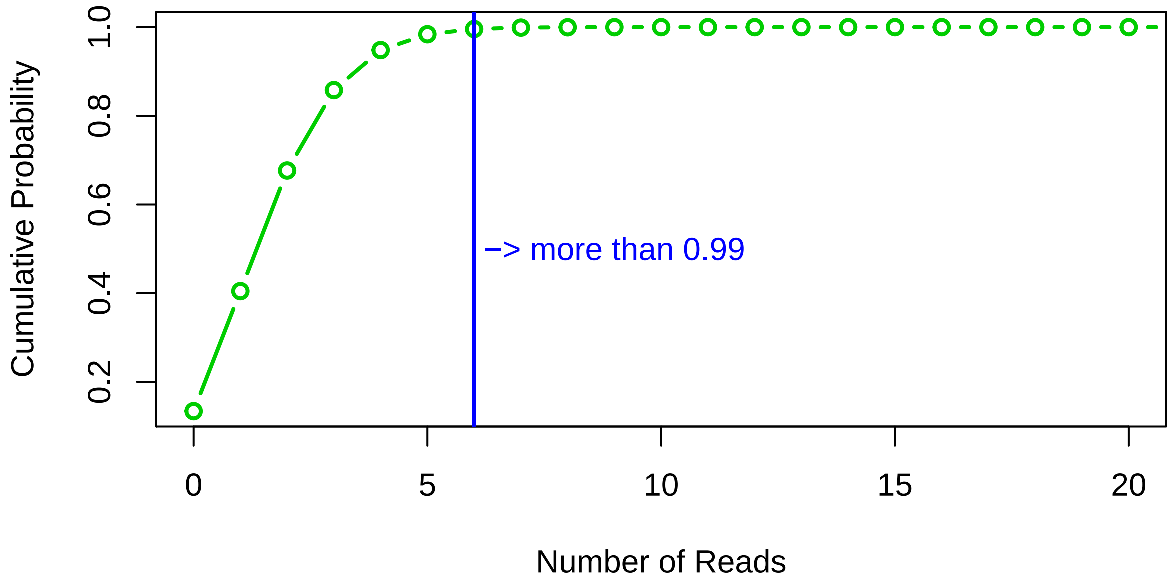

Figure S3: Cumulative distribution of sequencing errors for $p_e = 0.01$, with a vertical line denoting limit for which any greater or equal number of sequencing errors have cumulative probability greater than 0.99.

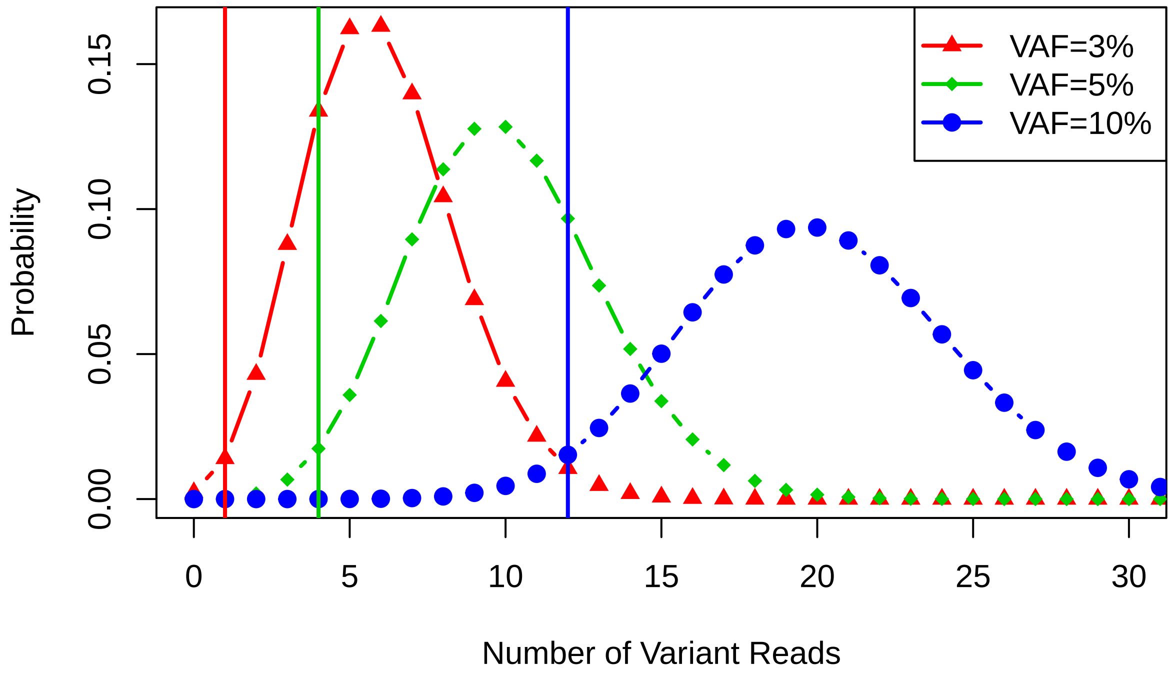

Figure S4: Binomial distribution of variant allele reads for three different variant allele frequencies, with a vertical line denoting limit for which any higher number of sequencing errors have cumulative probability greater than 0.99. VAF = variant allele frequency.

The iterative algorithm implementing aforementioned conditions is presented in the next section using R programming language.

Example R Script

- Variant allele frequency: $v_f = 0.1$.
- Sequencing error: $p_e = 0.01$.
- Limit of cumulative probability for sequencing errors: $p_{fp} = 0.001$.
- Limit of cumulative probability for variant reads: $p_{tp} = 0.999$.

```r
coverage.relax <- function(vaf,e,pfp,ptp,minvar=1,max_iterations=1000){
  coverage = 0
  vf = 0

  cov_aux = 5

  while(cov_aux < max_iterations){
    k = seq(0,cov_aux,by=1)
    e_binom = pbinom(k, cov_aux, e)

    se = 0
    for(e_id in 1:cov_aux){
      if(1 - e_binom[e_id] >= pfp){
        se = se + 1
      }
      else{
        break
      }
    }

    vf_binom = pbinom(k, cov_aux, vaf)
    if(se == 0){
      se = se + 1
    }

    if(1 - vf_binom[se] >= ptp && minvar <= se){
      vf = se
      coverage = cov_aux
      break
    }

    cov_aux = cov_aux + 1
  }
  c(coverage,vf)
}
```

```
## [1] "Minimum sequencing coverage depth and variant allele frequency"
```

```r
coverage.relax(vaf=0.1,e=0.01,pfp=0.001,ptp=0.999)
```

```
## [1] 175   7
```

```
## [1] "Minimum sequencing coverage depth and variant allele frequency"
```

```r
coverage.relax(vaf=0.1,e=0.01,pfp=0.001,ptp=0.999,minvar=10)
```

```
## [1] 300  10
```

Links

The OLGEN Coverage Limit Calculator can be accessed:

- Source codes - including python, R scripts and link to online application are available at: https://github.com/mvasinek/olgen-coverage-limit
